# Efficacy of *Sterculia diversifolia* Leaf Extracts: Volatile Compounds, Antioxidant and Anti-Inflammatory Activity, and Green Synthesis of Potential Antibacterial Silver Nanoparticles

**DOI:** 10.3390/plants11192492

**Published:** 2022-09-23

**Authors:** Ezz Al-Dein M. Al-Ramamneh, Ayoup M. Ghrair, Ashok K. Shakya, Khalid Y. Alsharafa, Khalid Al-Ismail, Samer Y. Al-Qaraleh, Jacek Mojski, Rajashri R. Naik

**Affiliations:** 1 Department of Agricultural Sciences, AL-Shouback University College, Al-Balqa Applied University, Maan 71911, Jordan; 2 Department of Water Resources and Environmental Management, Faculty of Agricultural Technology, Al-Balqa Applied University, Salt 19385, Jordan; 3 Pharmacological and Diagnostic Research Center, Department of Pharmaceutical Sciences, Faculty of Pharmacy, Al-Ahliyya Amman University, Amman 19328, Jordan; 4 Department of Biological Sciences, Faculty of Science, Mutah University, Mutah 61710, Jordan; 5 Department of Nutrition and Food Technology, Faculty of Agriculture, The University of Jordan, Amman 11942, Jordan; 6 Department of Pharmacology, Faculty of Medicine, Mut’ah University, Mutah 61710, Jordan; 7 Twój Swiat Jacek Mojski, Ulica Okrzei 39, 21-400 Lukow, Poland; 8 Pharmacological and Diagnostic Research Center, Faculty of Allied Medical Sciences, Al-Ahliyya Amman University, Amman 19328, Jordan

**Keywords:** silver nanoparticles, *Sterculia diversifolia*, antioxidant, antibacterial, anti-inflammatory, total phenolics, total flavonoids

## Abstract

*Sterculia diversifolia*, widely distributed in Jordan as an ornamental plant, is a synonoum for *Brachychiton populneus*. Phytochemical studies examining the volatile chemicals in *Sterculia diversifolia* leaves are limited, despite the rising demand for their numerous applications. Furthermore, it was only recently that a report described the friendly synthesis of silver nanoparticles (AgNPs) using aqueous extract derived from *Brachychiton populneus* leaves. Therefore, AgNPs were produced using either aqueous plant extracts (AgWPE) or ethanolic plant extracts (AgEPE), and Shimadzu GC-MS equipment was used to detect volatile compounds in the ethanolic leaf extracts. GC-MS profile of leaf ethanolic extracts of the Jordanian chemotypes of *S. diversifolia* revealed the existence of major components: (3β)-Lup-20(29)-en-3-ol acetate (30.97%) and 1-octadecyne (24.88). Other compounds are squalene (7.19%), germanicol (6.23), dl-α-tocopherol (5.24), heptacosane (4.41), phytol (3.54) and pentacosane (2.89). According to published studies, these reported chemicals have numerous uses, including as animal feed, vitamin precursors, possible eco-friendly herbicides, antioxidants, and anti-inflammatory agents. Aqueous extracts of *S. diversifolia* leaves had total phenolic of 5.33 mg GAE/g extract and flavonoid contents of 64.88 mg QE/g extract, respectively. The results indicated the contribution of phenolic and flavonoids to this plant’s anti-inflammatory and antioxidant properties. The reduction in AgNO_3_ to AgNPs using *S. diversifolia* leaf extracts was confirmed by the change in solution color from colorless to dark black. Further characterization was attempted by X-ray diffraction, Malvern zeta-sizer and scanning electron microscope. The efficacy of synthesized Ag nanoparticles using aqueous or ethanolic plant extract of *S. diversifolia* against the Gram-negative bacteria *Escherichia coli* and Gram-positive bacteria *Staphylococcus aureus* showed appreciable activity at 25 µg/mL concentration compared to the source plant extracts.

## 1. Introduction

*Sterculia diversifolia* is known as *Brachychiton* or kurrajong [1,2]. Numerous uses for the tree and its parts have been reported, including roasted seeds as coffee substitutes; many of its parts are also consumed as food sources in areas of Australia, and fodder for animals in times of drought [3]. The plant, which varies in size from being a small shrub to up to 20 m in height, was introduced in Jordan and is an attractive ornamental tree along roadsides. Although *Sterculia diversifolia* is a synonym for *Brachychiton populneus* according to the global plants database [4], variation based on the botanical examination of the seeds and follicles led some researchers to speculate that *Brachychiton* may be treated as an independent genus [5].

Metallic nanoparticles (MNPs) are increasingly used in industrial applications and commercial products [6]. Using MNPs in agriculture to more effectively utilize inputs such as fertilizers and pesticides results in better crop performance and promotes sustainable farming. Because they can be created in a variety of sizes and qualities, MNPs have become an important instrument not only in agriculture but also in the fields of medicine, cosmetics, food and environmental sciences [7,8]. A growing priority is the green synthesis of MNPs using plant extracts that can reduce metal ions to MNPs using eco-friendly ways. This is because the manufacture of MNPs using chemical methods has raised toxicity and environmental issues [8]. The production of engineered green MNPs could be achieved using plants as well as microorganisms [9,10]. Green synthesis of AgNPs was attempted using plant extracts [11]. AgNPs are special nanomaterials that can be used as antibacterial agents without harming human cells, and are helpful as a surgical technique for reducing infections [12,13]. Additionally, AgNPs are employed in the production of composite fibers and cosmetics [8]. Plant extracts, depending on extraction methods, were shown to have different levels of antioxidant activities because many phenolic compounds depending on their chemical-physiological nature need a reliable method to be freed from the covalent bonds in which they exist in plants [14]. Metal nanoparticles obtained from medicinal plant extracts such as rosemary have proven efficient as antioxidants in food technology [15]. MNPs have also been used efficiently as antimicrobial agents; an example is the use of AgNPs synthesized using *Salvia spinosa* plant extract [10]. The phytochemicals flavonoids, sterols, saponins, triterpenoids, phenols, tannins, and coumarins are reported to be abundant in *Sterculia diversifolia* extracts, many of which are helpful in the treatment of microbial infections, inflammations, hyperglycemia, cancer, and skin problems because they have antioxidant and antibacterial properties [1,5,16,17,18]. The domestication of the certified genotypes of *Sterculia diversifolia* grown in Jordan will have its influence on the bioconstituents of plant extracts. Therefore, the study of volatile compounds of *Brachychiton* grown in Jordan will provide information on the chemotype profiling of Jordanian *Sterculia diversifolia* and thus may increase information on the taxonomic status of the *Brachychiton* species grown in different regions worldwide. The synthesis of AgNPs from aqueous extract has been recently attempted from leaves of *B. populneus* collected from Lahore, Pakistan [19]. Therefore, *Sterculia diversifolia* genotypes grown in Jordan seems to be promising for the synthesis of silver nanoparticles which has the potential to gain attention for the ecologically sustainable synthesis of nanoparticles. The study also aimed to explore the ability of synthesized NPs to act as an antimicrobial agent against Gram-negative bacteria (*E. coli* ATCC 8739) and Gram-positive bacteria (*S. aureus* ATCC 6538).

## 2. Results and Discussion

### 2.1. Identification of Compounds

The identification of the constituents was based on computer matching against commercial, comparison of the linear retention indices (LRI) to a series of n-hydrocarbons, comparison of the retention times (Rt) to those of pure authentic samples, and use of a homemade library of mass spectra made up from pure substances and components of known oils, as well as MS literature data (Table 1 and Table 2). The major volatile compounds identified are (3β)-Lup-20(29)-en-3-ol acetate (30.97%) and 1-Octadecyne (24.88). Other compounds are squalene (7.19%), germanicol (6.23), dl-α-Tocopherol (5.24), heptacosane (4.41), Phytol (3.54), and pentacosane (2.89). The GC-MS identified about 85.4% of natural volatile compounds present in the extract and the other compounds were classified as unknown. 1-Octadecyne is considered a galactogogues, which could have potential use in feeding dairy cattle [20]. This compound was identified in the ethanolic extract of *Gliricidia maculata* [20] and a volatile component from *Momordica charantia* leaf ethanolic extract [21]. Phytol is a diterpene that is used in the synthesis of vitamin E and Vitamin K1 [22,23]. Pentacosane is an n-alkanes compound with potential uses as an eco-friendly type of herbicide [24]. Alpha-tocopherol is an antioxidant of the vitamin E family [25]. Heptacosane is a straight chain of alkane that was identified in the essential oils of *Centaurea sicana* [26]. Squalene molecules, known for nutritional and pharmaceutical uses, are sterols-precursor extracted from plants [27]. Germanicol is a triterpenoid that was identified in the methanolic extract of *Celtis sinensis* [28].

### 2.2. Synthesis of Silver Nanoparticles

The green synthesis experiment of AgNPs using *Sterculia diversifolia* plant extract revealed that the plant extract was capable of reducing the silver cation from ^+^1 valence to zero valence at pH 9 and 70 °C. The appearance of dark black color was an indicator of forming Ag-solid nanoparticles. Pirtarighat et al. [10] reported that AgNPs can be synthesized using toxic and harmful chemical reducing agents. However, using natural reducing agents from plant extracts is highly recommended. Moreover, Bhui and Misra [29] found that cellulosic derivatives from natural plant extracts can act as stabilizing and reducing agents. 

The constituents were identified by using GC-MS and represents the main components of the plant extract (Table 1). The hydrocarbon compounds such as phytol, tocopherol, and heptacosane play a major role in producing and stabilizing the AgNPs (Table 2). For instance, α-tocopherol acts as a reducing agent which is responsible for the reduction in Ag^+^ to Ag solid nanoparticles. Proudfoot et al. [30] reported that tocopherol is a major reducing agent, converting Cu^2+^ to Cu^+^. It has been suggested by Roopan et al. [31] that plant hydrocarbons such as heptacosane have a positive effect on the stabilization of AgNPs.

**Figure 1:**
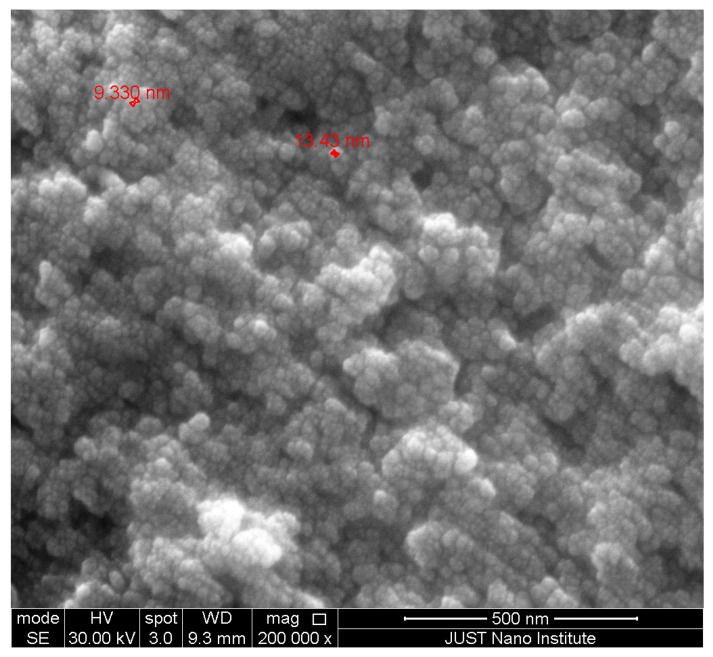
Green synthesis of AgEPE; size observed by SEM at 30 kV and 200,000× magnification.

**Figure 2:**
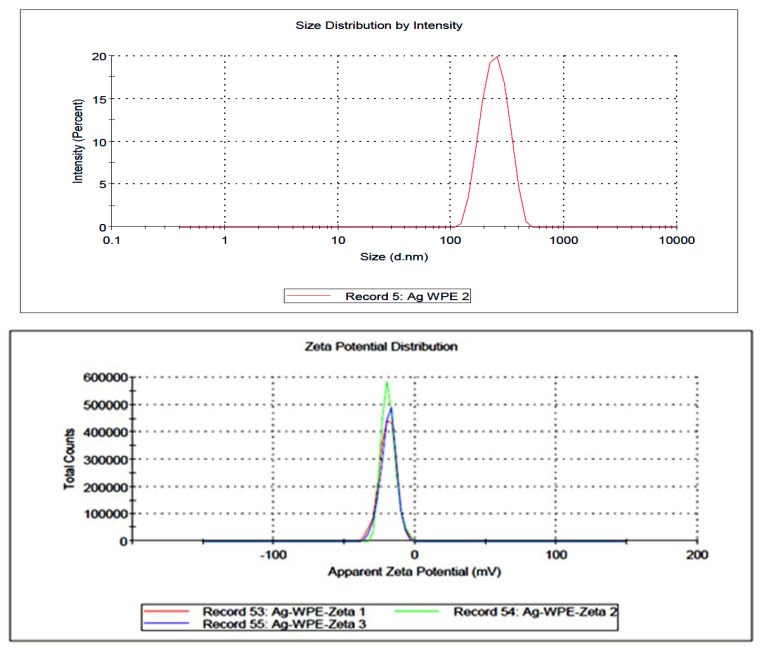
Size and charge of silver nanoparticles prepared using aqueous (AgWPE) extract as measured by zeta sizers.

**Figure 3:**
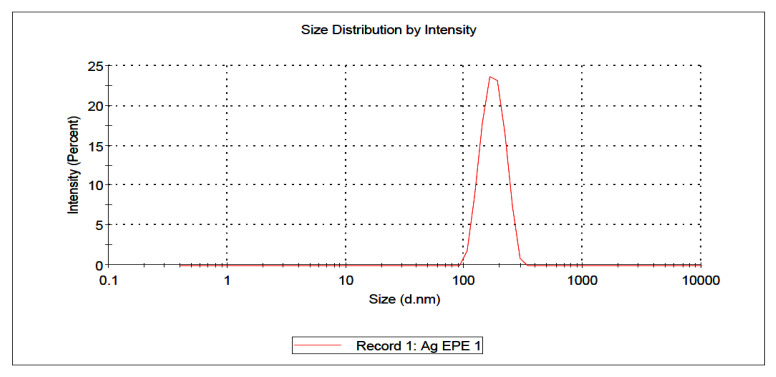

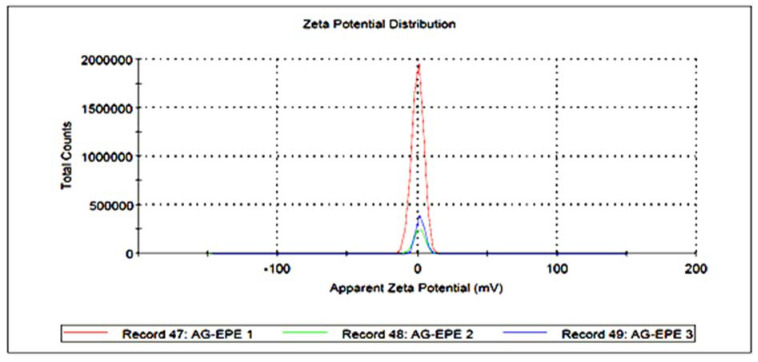
Size and charge of silver nanoparticles prepared using ethanolic (AgEPE) extract as measured by zeta sizers.

The SEM image confirmed that the particle size of AgEPE (AgNPs synthesized using ethanolic plant extract) is within the nano range, with an average particle size of around 15 nm (Figure 1). Moreover, particles are present in the form of single and agglomerated particles. Based on the SEM image, the particle shape tended to be spherical with smooth edges. The zeta sizers measure the size and charge of free as well as agglomerated particles. The data from the zeta sizer indicate that AgWPE are more stable than those using the ethanolic extract; this might be due to the negative charge (−19.0 ± 3.20 mV) on the particles which stabilizes the AgNPs (Figure 2 and Figure 3). The higher agglomeration in ethanolic plant extract is evident by the larger particle size distribution (304.4 nm) than in the aqueous extract of the plant (247.1 nm) (Table 3), where the reduction in the free energy of the particles in the ethanolic extract is most likely accompanied by size increment of the nanoparticle than in aqueous extract. The charge on AgEPE is close to 2.12 ± 1.84 mV, which facilitates the agglomeration. The increased charge of nanoparticles in the aqueous extract will probably be accompanied by higher repulsion of particles, leading to more electrostatic stability and which may explain the smaller size distribution [32].

**Figure 4:**
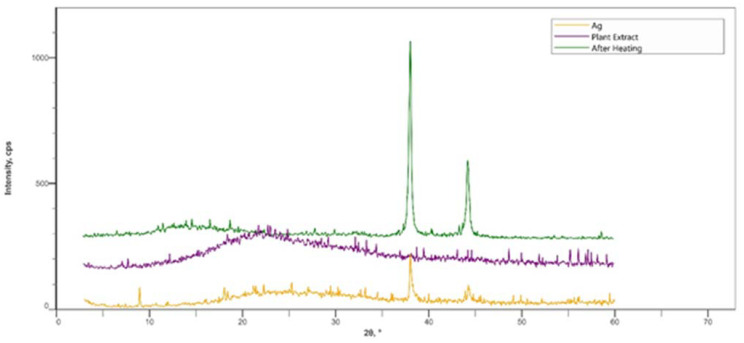
XRD patterns for random orientation powder of green synthesized AgNPs and dry powder of Ethanolic plant extract [(Ag): Synthesized AgNPs using Ethanol plant extract (AgEPE) at 30 °C, (After Heating): calcined Synthesized AgEPE at 250 °C, and (Plant extract): Ethanolic plant extract at 30 °C], * in x-axis stands for degree.

The X-ray diffractogram patterns revealed that the mineral content of the calcined synthesized nanoparticles powder is pure Silver (Figure 4, After heating). The XRD analysis showed three main peaks at 38.2°, 44.35°, and 58.7° 2θ with peaks intensity 791, 319, and 35 CPS, respectively. The XRD pattern agrees with [33], whereas the X-ray diffractogram patterns of the synthesized nanoparticles powder that dried at 30 °C showed six major peaks at 8.95°, 38.0°, 44.25°, 46.2°, 49.9°, and 59.45° 2θ with peaks intensity 89, 220, 100, 35, 59, and 66 CPS, respectively. In addition, the X-ray diffractogram patterns of the plant extract showed that no peaks were observed. The comparison between the XRD patterns before calcination and after calcination revealed that the main intense diffraction peak was shifted slightly to higher values of 2θ. The main peaks 2θ of AgNPs were shifted from 38.0° and 44.25° 2θ to 38.2° and 44.35° 2θ, respectively. Moreover, the peak at 8.95° 2θ does not appear after the calcination of AgNPs. One possible explanation is that organic compounds from the plant extracts are linked with AgNPs. Consequently, a shift in the AgNPs peaks 2θ occurred. In addition, after calcination, all the organic compounds were destroyed and resulting in a shift of the main X-ray diffraction peaks. These results are in agreement with Assis et al. [34]. They reported that the major intense diffraction peaks were shifted slightly to higher values of 2θ Ag-composites.

**Figure 5:**
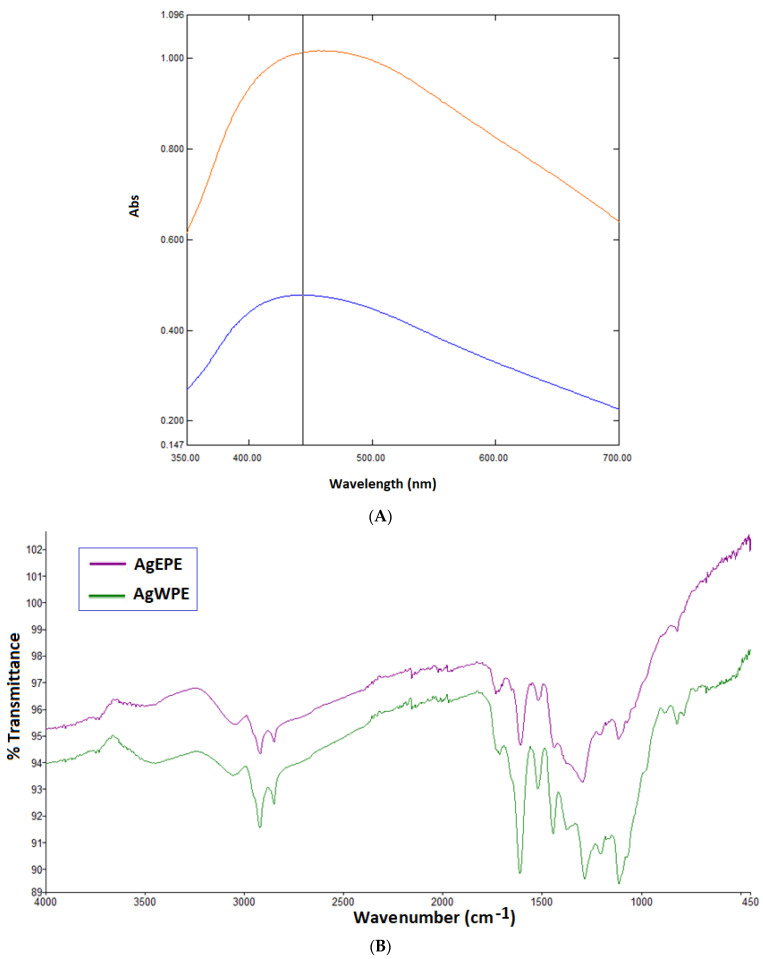
(**A**): UV-Vis absorption spectra of AgNPs synthesized using EPE (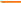) and WPE (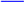); (**B**) FT-IR spectroscopic analysis of Synthesized AgNPs using EPE (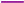) and WPE (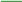).

UV-Vis Spectroscopic absorption band between 440 and 450 nm indicates the formation of AgNPs utilizing ethanolic ‘EPE’ and aqueous ‘WPE’ extract. There was no significant shift in the UV–Vis spectrum of the nanoparticles suspension even after 3 month, which revealed the nanoparticles’ stability (Figure 5A).

FTIR analysis of both synthesized nanoparticles AgEPE and AgWPE was carried out. The analysis of the FTIR spectrum of both types of obtained nanoparticles suggested extensive similarities between the samples. In particular, NPs are characterized by the O-H band at ~3400 cm^−1^, and the C-H stretching was observed between 2919 and 2859 cm^−1^. Moreover, C=C double-bond stretching at 1619 and 1528 cm^−1^ and C-O stretching at 1292 and 1113 cm^−1^ were detected in both samples (Figure 5B). Band corresponding to 1452 cm^−1^ is CH_3_ or CH_2_ asymmetric deformation. The bands at 1752 and 1650 cm^−1^ (merged with 1619 cm^−1^ C=C stretching) corresponded to C=O stretching (Figure 5B).

### 2.3. Biological Assays

#### 2.3.1. Total Phenolics and Flavonoids

The total phenolic and flavonoids in aqueous extracts of *S. diversifolia* leaves were 5.33 mg GAE/g extract, and 64.88 mg QE/g extract, respectively (Table 4). The results showed that ethanolic leaf extracts had total phenolics of 7.10 mg GAE/g extract and total flavonoids of 29.71 mg QE/g extract (Table 4). A group of seventeen flavonoids was earlier identified in 70% methanol extract of air-dried leafy cuttings collected from *Brachychiton* grown in Egypt [3]. Rjeibi et al. [35] studied the bioactive-phenolic substance profile of Tunisian *Brachychiton* and reported that major compounds were salvianolic acid B, syringic acid, caffeic acid, trans-ferulic acid, and quinic acid. The overall conclusion of these studies confirmed the role of phenolic and flavonoids in the antioxidant capacity as well as anti-inflammatory activity of this plant. 

#### 2.3.2. Antioxidant Activity

**Figure 6:**
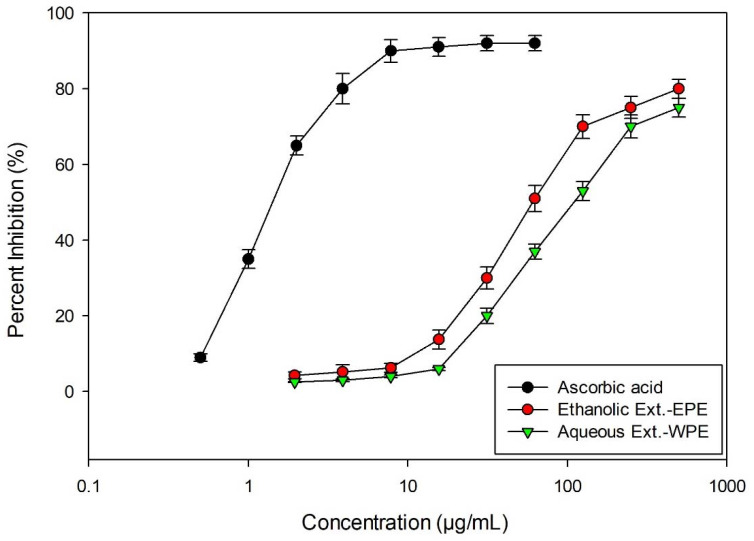
DPPH radical scavenging activity of ascorbic acid (standard), EPE and WPE. Plot showing concentration versus percentage inhibition.

An illustration of the antioxidant activity of the leaf extracts of *S. diversifolia* is shown in (Table 5, Figure 6). Ethanolic extract exhibited higher antioxidant activity (IC_50_ = 65.4 μg/mL) than plant aqueous extract (IC_50_ = 119 μg/mL). The results of the present study are encouraging, indicating that the extracts are capable of scavenging ~80% of DPPH radical at 750 μg/mL concentration, while ascorbic acid (standard antioxidant) is able to scavenge ~90% of DPPH radical at 12.0 μg/mL concentration. 

Rjeibi et al. [35] reported for Tunisian *Brachychiton* a higher antioxidant activity in methanolic extract rather than in aqueous extract of leaves. The antioxidant activity might be due to the presence of phenolics and flavonoids in plant extracts, and the presence of alpha tocopherol and other unidentified phytochemicals present in the extract. Furthermore, the synergetic effects of the various bio constituents of the extract, which in turn differ in their structures, polarities, and ability to dissolve in a certain solvent, will influence their antioxidant ability [36,37].

#### 2.3.3. Inhibition of Protein Denaturation

The test control represents 100% protein denaturation. The results are compared with diclofenac potassium (250 µg/mL). Diclofenac potassium is a nonsteroidal anti-inflammatory drug (NSAID) and could be beneficial to relieve individuals from pain and treat inflammation [38]. However, such compounds were also implicated to cause side and adverse effects in some patients [39]. The maximum percentage inhibition of protein denaturation at 250 µg/mL of diclofenac potassium, WPE, and EPE was 88.5, 79.6, and 70.7%, respectively (Table 6). Thus, the efficacy of WPE and EPE against protein denaturation was 89.9%, 79.9% that of the standard drug “diclofenac potassium”. Therefore, natural extracts such as WPE and EPE derived from *S. diversifolia* could substitute reference drug for patients with sensitivity to NSAID category. Germanicol identified in the current study from *S. diversifolia* extracts was reported to possibly have an anti-inflammatory effect [40]. The presence of anti-inflammatory and antioxidant mediators in the methanolic extracts of *B. populneus* was confirmed in a previous study [1]. Myricetin, rutin, and catechin are some of the significant components that have been found in the methanolic extracts of *B. populneus* and have been shown to have anti-inflammatory potential [1].

#### 2.3.4. Antimicrobial

The results showed that synthesized nanoparticles using aqueous (AgWPE) or ethanolic extracts (AgEPE) showed appreciable activity at 25 µg/mL concentration compared to the source plant extracts (Table 7). The larger zone of inhibition in AgWPE than AgEPE demonstrates that the antibacterial activity of AgWPE is stronger than AgEPE against both strains (Table 7). This may be explained by the fact that nanoparticles in aqueous extracts have a larger negative charge and a narrower size distribution than those in ethanolic extracts, indicating that AgWPE is more stable than AgEPE (Figure 2 and Figure 3, Table 3). According to reports, AgNPs cause bacterial cell membrane damage by releasing Ag ions that build up in the membrane and then react with proteins and thiol groups to produce free radicals, which slowly kill the bacteria [41,42,43].

#### 2.3.5. Photosynthetic Pigments and Proline Content

The leaf photosynthetic pigment contents are directly correlated to the photosynthetic activity. The pigment contents change with leaf maturation, senescence and also depend on environmental factors [44]. Three chlorophyll parameters include chlorophyll *a* (Chl *a*), chlorophyll *b* (Chl *b*), total Chl (Chl *a* + Chl *b*), and the ratio of Chl *a* and Chl *b* (Chl *a*/*b*) in *S. diversifolia* leaves (Table 8) showed the normal levels of these parameters and ratios [45]. Furthermore, the chlorophyll: carotenoids ratio range implicated healthy, non-stressed leaves that are not highly senescent [46]. The importance for measuring the photosynthetic pigment contents may be to infer some physiological signals that could be extensively employed in stress detection [47]. Moreover, chlorophyll parameters also provide an indication of nutritional status [48]. In addition, the accumulation of carotenoids is regarded to suppress the oxidation caused by oxidative stress [49].

Proline and soluble carbohydrates are accumulated by plants to control osmotic potential. One of the earliest signs of stress was thought to be the proline content in leaves. The low proline concentration of *S. diversifolia* leaves may rely on a variety of variables, including the type of stress and duration of exposure [50]. Therefore, the proline level in leaves indicates that *S. diversifolia* was not subjected to stress under the study circumstances. In leaves that are in the late stages of senescence, during drought stress, and during fluctuations in nutritional status, proline catabolism may be activated effectively [50]. Our results suggested that *S. diversifolia* is a rich source of bio constituents and its extracts under normal conditions of growth had the ability to reduce silver ions [51].

## 3. Materials and Methods

### 3.1. Sample Preparation

**Figure 7:**
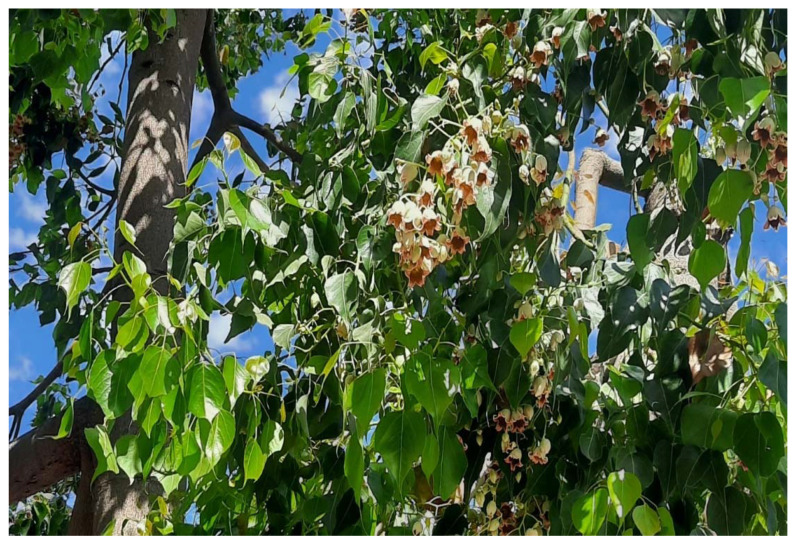
*Sterculia diversifolia* trees located in Amman, Jordan.

Leave samples were collected from *S. diversifolia* trees located in the Amman governate, Jordan (Latitude: 32.02126 N; Longitude: 35.84701 E, 1026 m above sea level) (Figure 7). The plant specimen was identified by Dr. Khaled Abu Laila, a senior botanist at the National Agricultural Research Center, and was also accredited by Greater Amman Municipality under the “guidelines of ornamental plants/booklet: ever green trees: page 62”. The fresh sample was air-dried at room temperature and ground using a domestic coffee mill.

### 3.2. Preparation of Ethanol and Water Extract

Forty grams of the ground herb was suspended in 1 L of ethanol, sonicated for 30 min at 40 °C using a sonicator, ISO LAB Germany, and then left for 24 h in the dark. The mixture was filtered using medium-rate filter paper and the volume was made up to 1 L with ethanol. The water extract followed the same procedure, except that the ground herb was immersed in 1 L of distilled water.

### 3.3. Synthesis of Silver Nanoparticles

A liter of 0.05 M silver nitrate (AgNO_3_) solution was prepared in distilled water and used as a source of silver. Twenty-five mL of herbal ethanolic extract was added dropwise to silver nitrate solution under continuously vigorous stirring using a magnetic stirrer. The mixture was heated to 70 °C for 30 min. The pH value of the reaction mixture was modified to 9 using a few drops of concentrated ammonium hydroxide. The mixture turned suddenly to dark black color. The suspension obtained was centrifuged (Hermle Z326, Labortechnik GmbH, Wehingen, Germany) at 10,000 rpm for 15 min. The separated AgNPs were washed three times with deionized water. The precipitated nanoparticles were suspended in 1 L of deionized water and stored in a cool and dark place for further characterization.

### 3.4. AgNPs Characterization: Determination of Particle Size, Shape, and Charge

Shimadzu’s (XRD-6000) X-ray diffractometer was used for X-ray diffraction to analyze the produced AgNPs utilizing a Cu. Kα. radiation source. Additionally, scanning electron microscope (SEM) pictures were obtained utilizing the Everhart–Thornley SE detector solid state (BSED), and CSEM-FEG inspect 50: field emission scanning electron microscope.

At 25 °C, photon correlation spectroscopy was utilized to measure particle size using Malvern Zeta-sizer Nano ZS^®^ equipment (Malvern, Model ZEN3600). The procedure included using a software and an instrument for dynamic light scattering from Malvern Instruments (Malvern, UK). The material was 50 times diluted in distilled water with a dispersant refractive index of 1.33. Samples were bubble-free for the accurate measurement of particle size. All measurements were made in triplicate at room temperature.

UV-Vis spectroscopic analysis was performed using standard methods. The UV-Vis spectra of the AgNPs were recorded using Shimadzu UV spectrophotometer (UV-1800, Shimadzu Corporation, Kyoto, Japan). The FTIR analysis was conducted on Perkin-Elmer FTIR Spectrum Two with UATR attachment (Perkin Elmer, USA). The samples were prepared by air-drying the purified nanoparticles and scanned on FTIR over the range of 4000–450 cm^−1^ at a resolution of 4 cm^−1^. The spectra recorded were plotted as transmittance (%) versus wave number (cm^−1^).

### 3.5. GC-MS Analysis of Ethanolic Extract

The procedure outlined by [52] using Shimadzu QP2020 GC-MS (Shimadzu Corporation, Kyoto, Japan) supplied with a split-split-less type injector, was utilized to analyze ethanol extracts and separate the volatile components using DB5 MS fused silica column. Besides the published data, every chemical component’s mass spectrum was compared to the corresponding reported spectrum for GC-MS including familiar libraries of ADAMS-2007 and NIST 2017. To ascertain the identified compound, a comparison was made between reported values and relative retention indices (RRI) in reference to *n*-alkanes (C_8_–C_35_).

### 3.6. Total Flavonoid Content

Using the technique outlined by [53], EPE and WPE was colorimetrically analyzed for their total flavonoid content. The flavonoid concentration was represented as mg quercetin equivalent/g of dry extract (mg QE/g).

### 3.7. Total Phenolic Content

Measurement of the total phenol concentration was feasible adopting the Folin–Ciocalteu method. Briefly, a mixture of 2.5 mL Folin–Ciocalteu reagent (2 N diluted tenfold) plus 2 mL of Na_2_CO_3_ solution were introduced to 0.5 mL of the extract. The mixture was given 15 min to stand at room temperature. Then, using methanol as a blank, the absorbance was determined at 765 nm. Then, total phenol content was given as mg gallic acid equivalent/g of dry extract (mg GAE/g). Each measurement was carried out three times [53].

### 3.8. DPPH Scavenging Activity

With certain modifications, the approach outlined by [53] was used. Briefly, 0.15 mL of each extract solution was combined with 0.150 mL of 0.2 mM DPPH^•^ solution (dissolved in MeOH). The solutions were dark incubated at room temperature for 30 min before being measured for absorbance at 517 nm in reference to a methanol blank using a Synergy HTC multimode reader. The following equation was used to determine the capacity to scavenge the DPPH^•^ radical

$$\mathrm{DPPH}\;\mathrm{scavenging}\;\mathrm{activity}\;\left(\%\right)=\left(\frac{\mathrm{absorbance}\;\mathrm{of}\;\mathrm{blank}-\mathrm{absorbance}\;\mathrm{of}\;\mathrm{extract}/\mathrm{std}}{\mathrm{absorbance}\;\mathrm{of}\;\mathrm{blank}}\right)\times 100$$

Using GraphPad Prism 6, non-linear regression analysis, scavenging activity and IC_50_ were determined in three replicates (GraphPad Software, San Diego, CA, USA).

### 3.9. Inhibition of Protein Denaturation

Inhibition of protein denaturation was conducted by adopting the method described by [54] with minor modification. Different solutions were prepared: Test, Test control, Product control, and standard solution. Each of the solutions was created in a pH 6.3 buffer. The samples underwent a 20 min incubation period at 37 °C, followed by a 15 min period at 50 °C. After cooling, the absorbance was determined at 416 nm with a Synergy HTC multimode reader. The formula used to compute the percentage inhibition of protein denaturation is,

$$\mathrm{Percent}\;\mathrm{Inhibition}=100-\left(\frac{\mathrm{absorbance}\;\mathrm{of}\;\mathrm{test}\;\mathrm{solution}-\mathrm{absorbance}\;\mathrm{of}\;\mathrm{product}\;\mathrm{control}}{\mathrm{absorbance}\;\mathrm{of}\;\mathrm{test}\;\mathrm{control}}\right)\times 100$$

### 3.10. Antimicrobial Activity

The ability of the extract, the silver nanoparticles, and Moxifloxacin (standard drug) to inhibit bacterial growth was examined using Gram-positive (*S. aureus* ATCC 6538) and Gram-negative (*E. coli* ATCC 8739) bacteria. In 9 cm plates with sterilized nutrient agar, the inhibition zones caused by the inoculum of the examined bacteria were measured. The 6 mm well were created using a sterilized 6 mm puncher. The wells were filled with 40 µL of test solutions (25 µg/mL) and the standard solution of moxifloxacin. The 5% DMSO was used as a control solution. Incubation of plates lasted for 24 h at 37 °C. Each experiment was carried out in duplicate. The average zone of inhibition is mentioned in Table 7.

### 3.11. Determination of Leaf Proline and Photosynthetic Pigments Content

The method demonstrated by [55] was adopted for the calculation of total free proline. Frozen leaves were subjected to extraction in a procedure that included centrifugation, boiling and cooling using prescribed reagents. Toluene was considered as the blank and reading were recorded at 520 nm. Proline concentration was determined using a calibration curve and expressed as mg proline g^−1^ FW.

Photosynthetic pigments were estimated spectrophotometrically. In brief, 20 mg of fresh leaf samples were extracted with 1 mL cold 80% acetone then incubated to 1 h in the dark and centrifugation (Hermle Z326, Labortechnik GmbH, Wehingen, Germany) at 13,000 rpm at 4 °C for 10 min. Finally, supernatant was read on 470, 645 and 663 nm. Chlorophylls (*a* and *b*) and carotenoids quantification were calculated according to [56] and [57].

## 4. Conclusions

The profile of volatile compounds in leaf ethanolic extracts of Jordanian *S. diversifolia* chemotype revealed the existence of major components: (3β)-Lup-20(29)-en-3-ol acetate (30.97%), 1-octadecyne (24.88), squalene (7.19%), germanicol (6.23), dl-α-tocopherol (5.24), heptacosane (4.41), phytol (3.54) and pentacosane (2.89). The volatile compounds identified in the current study from the extracts of *S. diversifolia* as well as the various phytochemicals reported from earlier studies, including flavonoids, sterols, saponins, triterpenoids, phenols, tannins, and coumarins, clearly demonstrated the antioxidant and antimicrobial activities of these phytoconstituents, which could be beneficial for the treatment of microbial infections, inflammations, hyperglycemic and skin disorders. AgNPs were efficient, eco-friendly, and simply produced using aqueous as well as ethanolic leaf extracts of *S. diversifolia*. The average particle size of AgNPs was around 15 nm with higher stability using aqueous rather than ethanolic extracts: probably due to the higher negative charge and smaller size distribution of the particles in aqueous extract than the ethanolic one. Synthesized AgNPs showed appreciable antibacterial activity against the Gram-negative *E. coli* and Gram-positive bacteria *S. aureus*, as shown by the agar well diffusion method. Overall, the wide array of bioactive components of *S. diversifolia* made it suitable for various nutritional, pharmaceutical, and biotechnological approaches, as demonstrated by the synthesis of AgNPs utilizing its extracts.

## Figures and Tables

**Table 1:** Constituents of *Sterculia diversifolia* plant ethanolic extract as revealed by GC-MS.

Peak	Ret. Time (min.)	Area%	Name	Ret. Index
**1**	23.407	24.88	1-Octadecyne	1856
**2**	24.13	1.96	Unknown	1878
**3**	27.442	3.54	Phytol	2104
**4**	32.428	2.89	Pentacosane	2492
**5**	32.799	4.19	Unknown	2524
**6**	34.762	4.41	Heptacosane	2690
**7**	36.31	7.19	Squalene	2804
**8**	42.546	5.24	dl-α-Tocopherol	3112
**9**	49.981	6.23	Germanicol	3327
**10**	51.569	30.97	(3β)-Lup-20(29)-en-3-ol acetate	3362

**Table 2:** Structure of the identified components of *Sterculia diversifolia* plant ethanolic extract.

Compound	Structure
Phytol	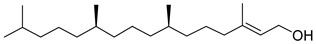
Squalene	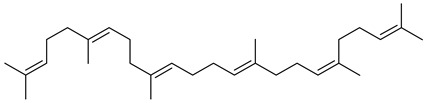
Germanicol	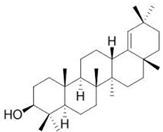
1-octadecyne	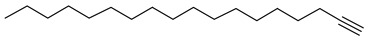
Heptacosane	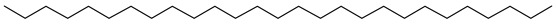
3β-Lup-20(29)-en-3-ol acetate	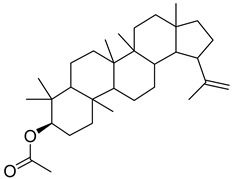
dl-α-tocopherol	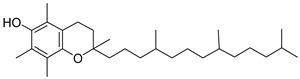

**Table 3:** Size distribution and zeta potential of AgNPs prepared using aqueous (WPE) or ethanolic (EPE) extracts.

Sample	Particle Size (nm) (Mean ± SD)	Zeta Potential (mV) (Mean ± SE)
AgWPE	247.1 ± 3.56	−19.0 ± 3.20
AgEPE	304.4 ± 9.61	2.12 ± 1.84

**Table 4:** Total phenolics and flavonoids in aqueous and ethanolic extracts of *S. diversifolia* leaves.

Sample	Total Phenolics (mg GAE/g Extract)	Total Flavonoids (mg QE/g Extract)
WPE	5.33 ± 0.01	64.88 ± 1.26
EPE	7.10 ± 0.03	29.71 ± 0.87

**Table 5:** In vitro DPPH radical scavenging: IC50 values were calculated for water and ethanol leaf extracts to scavenge DPPH free radicals. * (*n* = 3).

Sample	IC_50_ (µg/mL)
	DPPH Radical Activity *
WPE	119.0 ± 1.25
EPE	65.4 ± 1.02
Ascorbic Acid (50% EtOH)	1.25 ± 0.05

**Table 6:** Effects of water and ethanolic extract of *S. diversifolia* on protein denaturation, * (*n* = 3).

Sample *	% Protein Inhibition IC_50_ (µg/mL) (Mean ± SD)
WPE	95.5 ± 7.4
EPE	115.1 ± 8.8
Diclofenac Potassium	51.5 ± 5.2

**Table 7:** Antibacterial activity of the extracts and synthesized silver nanoparticles (AgNPs) against *E.coli* and *Staphylococcus aureus*.

	Inhibition Zone (mm)	
Sample Name	*E. coli*	*S. aureus*
WPE	8	8
EPE	9	8
AgWPE	18	18
AgEPE	16	15
Moxifloxacin (25 µg/mL)	30	52

*n* = 2 measurements, diameter of well = 6 mm.

**Table 8:** Chlorophyll *a*, chlorophyll *b*, carotenoids and proline content of *S. diversifolia* leaves, * (*n* = 3).

Measurement *	Content
Chlorophyll *a* (mg g^−1^ FW)	0.805495 ± 0.012062
Chlorophyll *b* (mg g^−1^ FW)	0.31829 ± 0.012019
Total chlorophyll (mg g^−1^ FW)	1.143893 ± 0.017276
Carotenoids (mg g^−1^ FW)	0.298165 ± 0.006863
Chlorophyll *a*:*b*	2.534345 ± 0.102977
Chlorophyll: carotenoids	3.83719 ± 0.037265
Proline (mg g^−1^ FW)	10.02667 ± 0.169967

## Data Availability

The data used are included in the manuscript.
